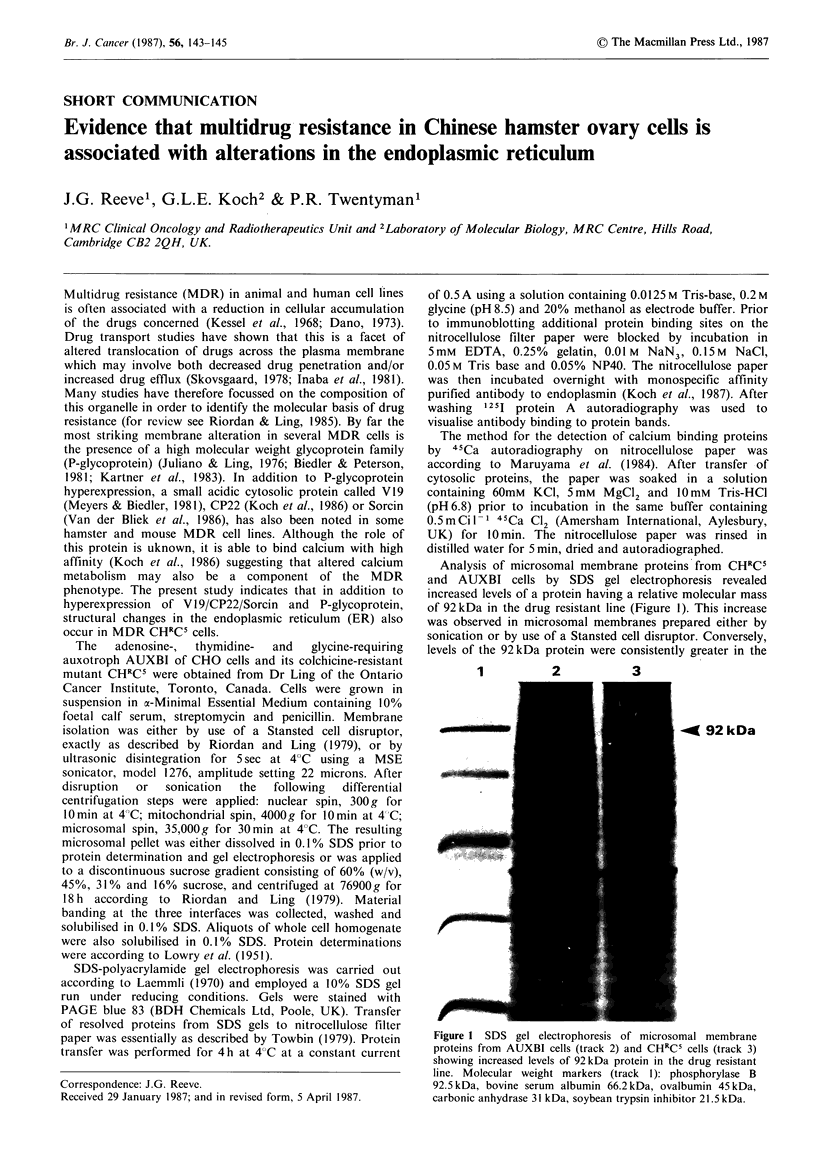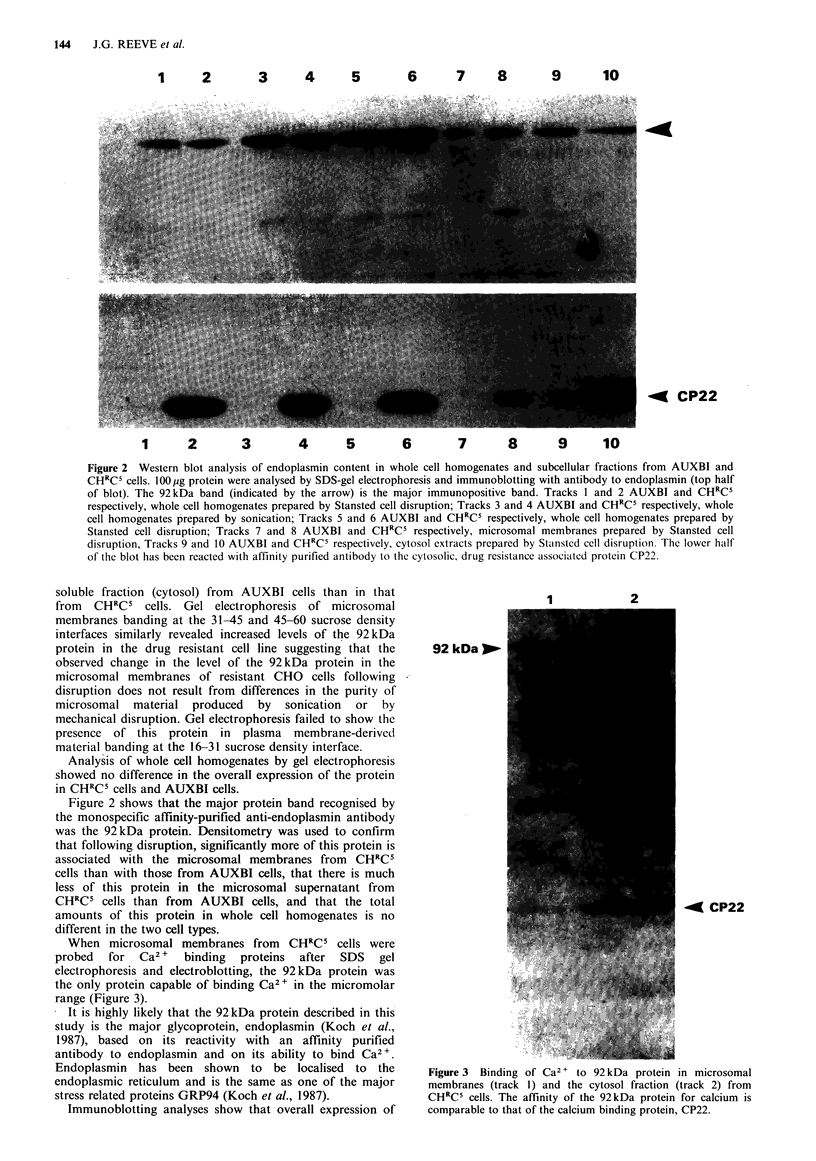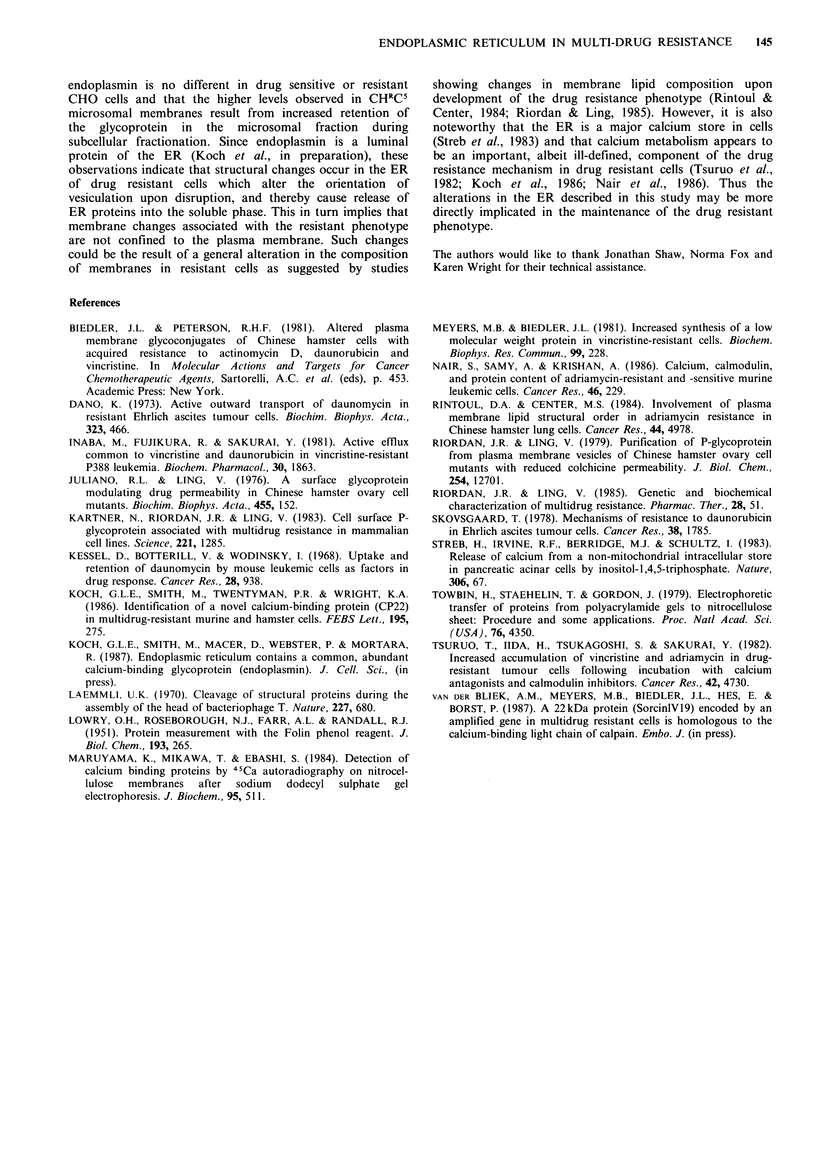# Evidence that multidrug resistance in Chinese hamster ovary cells is associated with alterations in the endoplasmic reticulum.

**DOI:** 10.1038/bjc.1987.173

**Published:** 1987-08

**Authors:** J. G. Reeve, G. L. Koch, P. R. Twentyman

**Affiliations:** MRC Clinical Oncology and Radiotherapeutics Unit, MRC Centre, Cambridge, UK.

## Abstract

**Images:**


Br. J. Cancer (1987), 56, 143–145 © The Macmillan Press Ltd., 1987

SHORT COMMUNICATION

### Evidence that multidrug resistance in Chinese hamster ovary cells is associated with alterations in the endoplasmic reticulum

J.G. Reeve[1], G.L.E. Koch[2] & P.R. Twentyman[1]

[1]*MRC Clinical Oncology and Radiotherapeutics Unit and* [2]*Laboratory of Molecular Biology, MRC Centre, Hills Road, Cambridge CB2 2QH, UK.*

Multidrug resistance (MDR) in animal and human cell lines is often associated with a reduction in cellular accumulation of the drugs concerned (Kessel *et al.*, 1968; Dano, 1973). Drug transport studies have shown that this is a facet of altered translocation of drugs across the plasma membrane which may involve both decreased drug penetration and/or increased drug efflux (Skovsgaard, 1978; Inaba *et al.*, 1981). Many studies have therefore focussed on the composition of this organelle in order to identify the molecular basis of drug resistance (for review see Riordan & Ling, 1985). By far the most striking membrane alteration in several MDR cells is the presence of a high molecular weight glycoprotein family (P-glycoprotein) (Juliano & Ling, 1976; Biedler & Peterson, 1981; Kartner *et al.*, 1983). In addition to P-glycoprotein hyperexpression, a small acidic cytosolic protein called V19 (Meyers & Biedler, 1981), CP22 (Koch *et al.*, 1986) or Sorcin (Van der Bliek *et al.*, 1986), has also been noted in some hamster and mouse MDR cell lines. Although the role of this protein is uknown, it is able to bind calcium with high affinity (Koch *et al.*, 1986) suggesting that altered calcium metabolism may also be a component of the MDR phenotype. The present study indicates that in addition to hyperexpression of V19/CP22/Sorcin and P-glycoprotein, structural changes in the endoplasmic reticulum (ER) also occur in MDR $CH^RC^5$ cells.

The adenosine-, thymidine- and glycine-requiring auxotroph AUXBI of CHO cells and its colchicine-resistant mutant $CH^RC^5$ were obtained from Dr Ling of the Ontario Cancer Institute, Toronto, Canada. Cells were grown in suspension in α-Minimal Essential Medium containing 10% foetal calf serum, streptomycin and penicillin. Membrane isolation was either by use of a Stansted cell disruptor, exactly as described by Riordan and Ling (1979), or by ultrasonic disintegration for 5 sec at 4°C using a MSE sonicator, model 1276, amplitude setting 22 microns. After disruption or sonication the following differential centrifugation steps were applied: nuclear spin, 300*g* for 10 min at 4°C; mitochondrial spin, 4000*g* for 10 min at 4°C; microsomal spin, 35,000*g* for 30 min at 4°C. The resulting microsomal pellet was either dissolved in 0.1% SDS prior to protein determination and gel electrophoresis or was applied to a discontinuous sucrose gradient consisting of 60% (w/v), 45%, 31% and 16% sucrose, and centrifuged at 76900*g* for 18 h according to Riordan and Ling (1979). Material banding at the three interfaces was collected, washed and solubilised in 0.1% SDS. Aliquots of whole cell homogenate were also solubilised in 0.1% SDS. Protein determinations were according to Lowry *et al.* (1951).

SDS-polyacrylamide gel electrophoresis was carried out according to Laemmli (1970) and employed a 10% SDS gel run under reducing conditions. Gels were stained with PAGE blue 83 (BDH Chemicals Ltd, Poole, UK). Transfer of resolved proteins from SDS gels to nitrocellulose filter paper was essentially as described by Towbin (1979). Protein transfer was performed for 4 h at 4°C at a constant current of 0.5 A using a solution containing 0.0125 M Tris-base, 0.2 M glycine (pH 8.5) and 20% methanol as electrode buffer. Prior to immunoblotting additional protein binding sites on the nitrocellulose filter paper were blocked by incubation in 5 mM EDTA, 0.25% gelatin, 0.01 M $NaN_3$, 0.15 M NaCl, 0.05 M Tris base and 0.05% NP40. The nitrocellulose paper was then incubated overnight with monospecific affinity purified antibody to endoplasmin (Koch *et al.*, 1987). After washing $^{125}I$ protein A autoradiography was used to visualise antibody binding to protein bands.

The method for the detection of calcium binding proteins by $^{45}Ca$ autoradiography on nitrocellulose paper was according to Maruyama *et al.* (1984). After transfer of cytosolic proteins, the paper was soaked in a solution containing 60mM KCl, 5 mM $MgCl_2$ and 10 mM Tris-HCl (pH 6.8) prior to incubation in the same buffer containing 0.5 mCi $l^{-1}$ $^{45}Ca$ $Cl_2$ (Amersham International, Aylesbury, UK) for 10 min. The nitrocellulose paper was rinsed in distilled water for 5 min, dried and autoradiographed.

Analysis of microsomal membrane proteins from $CH^RC^5$ and AUXBI cells by SDS gel electrophoresis revealed increased levels of a protein having a relative molecular mass of 92 kDa in the drug resistant line (Figure 1). This increase was observed in microsomal membranes prepared either by sonication or by use of a Stansted cell disruptor. Conversely, levels of the 92 kDa protein were consistently greater in the

**Figure 1** SDS gel electrophoresis of microsomal membrane proteins from AUXBI cells (track 2) and $CH^RC^5$ cells (track 3) showing increased levels of 92 kDa protein in the drug resistant line. Molecular weight markers (track 1): phosphorylase B 92.5 kDa, bovine serum albumin 66.2 kDa, ovalbumin 45 kDa, carbonic anhydrase 31 kDa, soybean trypsin inhibitor 21.5 kDa.

Correspondence: J.G. Reeve.
Received 29 January 1987; and in revised form, 5 April 1987.

**Figure 2** Western blot analysis of endoplasmin content in whole cell homogenates and subcellular fractions from AUXBI and $CH^RC^5$ cells. 100 $\mu$g protein were analysed by SDS-gel electrophoresis and immunoblotting with antibody to endoplasmin (top half of blot). The 92 kDa band (indicated by the arrow) is the major immunopositive band. Tracks 1 and 2 AUXBI and $CH^RC^5$ respectively, whole cell homogenates prepared by Stansted cell disruption; Tracks 3 and 4 AUXBI and $CH^RC^5$ respectively, whole cell homogenates prepared by sonication; Tracks 5 and 6 AUXBI and $CH^RC^5$ respectively, whole cell homogenates prepared by Stansted cell disruption; Tracks 7 and 8 AUXBI and $CH^RC^5$ respectively, microsomal membranes prepared by Stansted cell disruption, Tracks 9 and 10 AUXBI and $CH^RC^5$ respectively, cytosol extracts prepared by Stansted cell disruption. The lower half of the blot has been reacted with affinity purified antibody to the cytosolic, drug resistance associated protein CP22.

soluble fraction (cytosol) from AUXBI cells than in that from $CH^RC^5$ cells. Gel electrophoresis of microsomal membranes banding at the 31–45 and 45–60 sucrose density interfaces similarly revealed increased levels of the 92 kDa protein in the drug resistant cell line suggesting that the observed change in the level of the 92 kDa protein in the microsomal membranes of resistant CHO cells following disruption does not result from differences in the purity of microsomal material produced by sonication or by mechanical disruption. Gel electrophoresis failed to show the presence of this protein in plasma membrane-derived material banding at the 16–31 sucrose density interface.

Analysis of whole cell homogenates by gel electrophoresis showed no difference in the overall expression of the protein in $CH^RC^5$ cells and AUXBI cells.

Figure 2 shows that the major protein band recognised by the monospecific affinity-purified anti-endoplasmin antibody was the 92 kDa protein. Densitometry was used to confirm that following disruption, significantly more of this protein is associated with the microsomal membranes from $CH^RC^5$ cells than with those from AUXBI cells, that there is much less of this protein in the microsomal supernatant from $CH^RC^5$ cells than from AUXBI cells, and that the total amounts of this protein in whole cell homogenates is no different in the two cell types.

When microsomal membranes from $CH^RC^5$ cells were probed for $Ca^{2+}$ binding proteins after SDS gel electrophoresis and electroblotting, the 92 kDa protein was the only protein capable of binding $Ca^{2+}$ in the micromolar range (Figure 3).

It is highly likely that the 92 kDa protein described in this study is the major glycoprotein, endoplasmin (Koch *et al.*, 1987), based on its reactivity with an affinity purified antibody to endoplasmin and on its ability to bind $Ca^{2+}$. Endoplasmin has been shown to be localised to the endoplasmic reticulum and is the same as one of the major stress related proteins GRP94 (Koch *et al.*, 1987).

Immunoblotting analyses show that overall expression of

**Figure 3** Binding of $Ca^{2+}$ to 92 kDa protein in microsomal membranes (track 1) and the cytosol fraction (track 2) from $CH^RC^5$ cells. The affinity of the 92 kDa protein for calcium is comparable to that of the calcium binding protein, CP22.

endoplasmin is no different in drug sensitive or resistant CHO cells and that the higher levels observed in $CH^RC^5$ microsomal membranes result from increased retention of the glycoprotein in the microsomal fraction during subcellular fractionation. Since endoplasmin is a luminal protein of the ER (Koch *et al.*, in preparation), these observations indicate that structural changes occur in the ER of drug resistant cells which alter the orientation of vesiculation upon disruption, and thereby cause release of ER proteins into the soluble phase. This in turn implies that membrane changes associated with the resistant phenotype are not confined to the plasma membrane. Such changes could be the result of a general alteration in the composition of membranes in resistant cells as suggested by studies showing changes in membrane lipid composition upon development of the drug resistance phenotype (Rintoul & Center, 1984; Riordan & Ling, 1985). However, it is also noteworthy that the ER is a major calcium store in cells (Streb *et al.*, 1983) and that calcium metabolism appears to be an important, albeit ill-defined, component of the drug resistance mechanism in drug resistant cells (Tsuruo *et al.*, 1982; Koch *et al.*, 1986; Nair *et al.*, 1986). Thus the alterations in the ER described in this study may be more directly implicated in the maintenance of the drug resistant phenotype.

The authors would like to thank Jonathan Shaw, Norma Fox and Karen Wright for their technical assistance.

### References

BIEDLER, J.L. & PETERSON, R.H.F. (1981). Altered plasma membrane glycoconjugates of Chinese hamster cells with acquired resistance to actinomycin D, daunorubicin and vincristine. In *Molecular Actions and Targets for Cancer Chemotherapeutic Agents*, Sartorelli, A.C. *et al.* (eds), p. 453. Academic Press: New York.

DANO, K. (1973). Active outward transport of daunomycin in resistant Ehrlich ascites tumour cells. *Biochim. Biophys. Acta.*, **323**, 466.

INABA, M., FUJIKURA, R. & SAKURAI, Y. (1981). Active efflux common to vincristine and daunorubicin in vincristine-resistant P388 leukemia. *Biochem. Pharmacol.*, **30**, 1863.

JULIANO, R.L. & LING, V. (1976). A surface glycoprotein modulating drug permeability in Chinese hamster ovary cell mutants. *Biochim. Biophys. Acta.*, **455**, 152.

KARTNER, N., RIORDAN, J.R. & LING, V. (1983). Cell surface P-glycoprotein associated with multidrug resistance in mammalian cell lines. *Science*, **221**, 1285.

KESSEL, D., BOTTERILL, V. & WODINSKY, I. (1968). Uptake and retention of daunomycin by mouse leukemic cells as factors in drug response. *Cancer Res.*, **28**, 938.

KOCH, G.L.E., SMITH, M., TWENTYMAN, P.R. & WRIGHT, K.A. (1986). Identification of a novel calcium-binding protein (CP22) in multidrug-resistant murine and hamster cells. *FEBS Lett.*, **195**, 275.

KOCH, G.L.E., SMITH, M., MACER, D., WEBSTER, P. & MORTARA, R. (1987). Endoplasmic reticulum contains a common, abundant calcium-binding glycoprotein (endoplasmin). *J. Cell. Sci.*, (in press).

LAEMMLI, U.K. (1970). Cleavage of structural proteins during the assembly of the head of bacteriophage T. *Nature*, **227**, 680.

LOWRY, O.H., ROSEBOROUGH, N.J., FARR, A.L. & RANDALL, R.J. (1951). Protein measurement with the Folin phenol reagent. *J. Biol. Chem.*, **193**, 265.

MARUYAMA, K., MIKAWA, T. & EBASHI, S. (1984). Detection of calcium binding proteins by $^{45}Ca$ autoradiography on nitrocellulose membranes after sodium dodecyl sulphate gel electrophoresis. *J. Biochem.*, **95**, 511.

MEYERS, M.B. & BIEDLER, J.L. (1981). Increased synthesis of a low molecular weight protein in vincristine-resistant cells. *Biochem. Biophys. Res. Commun.*, **99**, 228.

NAIR, S., SAMY, A. & KRISHAN, A. (1986). Calcium, calmodulin, and protein content of adriamycin-resistant and -sensitive murine leukemic cells. *Cancer Res.*, **46**, 229.

RINTOUL, D.A. & CENTER, M.S. (1984). Involvement of plasma membrane lipid structural order in adriamycin resistance in Chinese hamster lung cells. *Cancer Res.*, **44**, 4978.

RIORDAN, J.R. & LING, V. (1979). Purification of P-glycoprotein from plasma membrane vesicles of Chinese hamster ovary cell mutants with reduced colchicine permeability. *J. Biol. Chem.*, **254**, 12701.

RIORDAN, J.R. & LING, V. (1985). Genetic and biochemical characterization of multidrug resistance. *Pharmac. Ther.*, **28**, 51.

SKOVSGAARD, T. (1978). Mechanisms of resistance to daunorubicin in Ehrlich ascites tumour cells. *Cancer Res.*, **38**, 1785.

STREB, H., IRVINE, R.F., BERRIDGE, M.J. & SCHULTZ, I. (1983). Release of calcium from a non-mitochondrial intracellular store in pancreatic acinar cells by inositol-1,4,5-triphosphate. *Nature*, **306**, 67.

TOWBIN, H., STAEHELIN, T. & GORDON, J. (1979). Electrophoretic transfer of proteins from polyacrylamide gels to nitrocellulose sheet: Procedure and some applications. *Proc. Natl Acad. Sci. (USA)*, **76**, 4350.

TSURUO, T., IIDA, H., TSUKAGOSHI, S. & SAKURAI, Y. (1982). Increased accumulation of vincristine and adriamycin in drug-resistant tumour cells following incubation with calcium antagonists and calmodulin inhibitors. *Cancer Res.*, **42**, 4730.

VAN DER BLIEK, A.M., MEYERS, M.B., BIEDLER, J.L., HES, E. & BORST, P. (1987). A 22kDa protein (SorcinlV19) encoded by an amplified gene in multidrug resistant cells is homologous to the calcium-binding light chain of calpain. *Embo. J.* (in press).